# Long-term stroke and major bleeding risk in patients with non-valvular atrial fibrillation: A comparative analysis between non-vitamin K antagonist oral anticoagulants and warfarin using a clinical data warehouse

**DOI:** 10.3389/fneur.2023.1058781

**Published:** 2023-01-26

**Authors:** Hancheol Lee, Jung Hwa Hong, Kwon-Duk Seo

**Affiliations:** ^1^Department of Cardiology, National Health Insurance Service Ilsan Hospital, Goyang, Republic of Korea; ^2^Institute of Health Insurance and Clinical Research, National Health Insurance Service Ilsan Hospital, Goyang, Republic of Korea; ^3^Department of Neurology, National Health Insurance Service Ilsan Hospital, Goyang, Republic of Korea; ^4^Department of Neurology, Graduate School of Medicine, Kangwon National University, Chuncheon, Republic of Korea

**Keywords:** atrial fibrillation, NOAC, warfarin, ischemic stroke, intracranial hemorrhage, gastrointestinal bleeding

## Abstract

**Introduction:**

Non-vitamin K antagonist oral anticoagulants (NOACs) has been the drug of choice for preventing ischemic stroke in patients with atrial fibrillation (AF) since 2014. Many studies based on claim data revealed that NOACs had comparable effect to warfarin in preventing ischemic stroke with fewer hemorrhagic side effects. We analyzed the difference in clinical outcomes according to the drugs in patients with AF based on the clinical data warehouse (CDW).

**Methods:**

We extracted data of patients with AF from our hospital's CDW and obtained clinical information including test results. All claim data of the patients were extracted from National Health Insurance Service, and dataset was constructed by combining it with CDW data. Separately, another dataset was constructed with patients who could obtain sufficient clinical information from the CDW. The patients were divided NOAC and warfarin groups. The occurrence of ischemic stroke, intracranial hemorrhage, gastrointestinal bleeding, and death were confirmed as clinical outcome. The factors influencing the risk of clinical outcomes were analyzed.

**Results:**

The patients who were diagnosed AF between 2009 and 2020 were included in the dataset construction. In the combined dataset, 858 patients were treated with warfarin, 2,343 patients were treated with NOACs. After the diagnosis of AF, the incidence of ischemic stroke during follow-up was 199 (23.2%) in the warfarin group, 209 (8.9%) in the NOAC group. Intracranial hemorrhage occurred in 70 patients (8.2%) among the warfarin group, 61 (2.6%) of the NOAC group. Gastrointestinal bleeding occurred in 69 patients (8.0%) in the warfarin group, 78 patients (3.3%) in the NOAC group. NOAC's hazard ratio (HR) of ischemic stroke was 0.479 (95% CI 0.39–0.589, *p* < 0.0001), HR of intracranial hemorrhage was 0.453 (95% CI 0.31–0.664, *p* < 0.0001), and HR of gastrointestinal bleeding was 0.579 (95% CI 0.406–0.824, *p* = 0.0024). In the dataset constructed using only CDW, the NOAC group also had a lower risk of ischemic stroke and intracranial hemorrhage than warfarin group.

**Conclusions:**

In this CDW based study, NOACs are more effective and safer than warfarin in patients with AF even with long-term follow-up. NOACs should be used to prevent ischemic stroke in patients with AF

## Introduction

Atrial fibrillation is the most dangerous comorbidity of ischemic stroke as it causes more than five-fold increase in the risk of developing ischemic stroke compared to individuals without atrial fibrillation (1). The prevalence of atrial fibrillation is ~1–1.6% (2), and it increases to ~8.15% in patients at least 80 years of age (3). In Asian countries where the elderly population is growing rapidly, the prevalence is also increasing rapidly (3, 4). Furthermore, atrial fibrillation occurs in ~15–20% of patients with ischemic stroke (5). Approximately 36–40% of patients with ischemic stroke aged ≥80 years have atrial fibrillation (6, 7). Therefore, medical treatment to prevent ischemic stroke in patients with atrial fibrillation is essential to reduce the incidence of ischemic stroke.

In the past, warfarin, a vitamin K antagonist, was the only anticoagulant treatment used to prevent ischemic stroke secondary to atrial fibrillation. However, achieving the target blood concentration of warfarin that provides anticoagulant effects is challenging and may result in hemorrhagic side effects that are more common in Asians (8, 9). Several randomized controlled trials (RCTs) have reported that new non-vitamin K oral anticoagulants (NOACs) have similar efficacy in ischemic stroke prevention and fewer side effects (including bleeding) than warfarin (10–13). As NOACs were first recommended in 2014, the current guidelines suggest that they be used as an anticoagulant in patients with atrial fibrillation instead of warfarin unless contraindications to NOACs exist (14).

Several studies based on claim data comparing the effectiveness and safety of NOACs to those of warfarin have reported similar results as the previous RCTs (15, 16). However, since these studies were conducted not long after NOAC was recommended, the long-term follow-up results were unknown. Claim data studies have the advantage of analyzing several individuals based on big data; however, they cannot accurately reflect patient test results or clinical information such as weight, alcohol intake, and smoking status. As hospital electronic medical records (EMR) contain such data, clinical studies have been conducted using EMR. EMR-based studies are designed similarly to cohort studies and can include long-term follow-up data (17). In addition, although retrospective, the data can be analyzed without selection bias. However, some EMR-based studies are limited to a single institution. In this study, dataset combining our hospital's EMR-based clinical data warehouse (CDW) and claim data including the whole medical record was also constructed in order to compensate for the disadvantages of using data from a single institution. This study aimed to compare and analyze the risk of developing ischemic stroke and hemorrhagic side effects during long-term follow-up in patients with atrial fibrillation treated with warfarin to those of patients treated with NOACs.

## Methods

### Data source and study population

Data were extracted from the CDW that was established using the medical records of the National Health Insurance Service Ilsan Hospital. All data used in this study were dated between 2009 and 2020. Patients aged ≥20 years who were diagnosed with International Classification of Diseases 10th revision (ICD-10) code 148 (atrial fibrillation) as a principal diagnosis or a first secondary diagnosis who had been treated more than once were included in this study. Patients with rheumatic mitral valve diseases (ICD-10 code I05) and those who underwent heart valve surgery (ICD-10 code Z95.2-4), which is not included in the indications of NOACs, were excluded from the study.

The CDW+C data was constructed by combining patient data extracted from the CDW with whole claim data. To investigate the occurrences of ischemic stroke or hemorrhagic side effects after a diagnosis of atrial fibrillation, patients diagnosed with atrial fibrillation prior to 2009 (when the CDW was established) were excluded. As the medication administration records were available within the claim data, patients whose anticoagulant medication was changed during the follow-up period were excluded from this analysis. The patients with no anticoagulant were excluded. The follow-up period was from the time of diagnosis of atrial fibrillation to December 31, 2020.

The CDW-O data were constructed by selecting patients with sufficient clinical information. Patients with no available data regarding medications and those who were administered anticoagulant medications for <30 days were excluded from the study. The patients who were administered antiplatelet medication only were excluded. Patients diagnosed with concomitant atrial fibrillation and ischemic stroke were excluded from the analysis. Patients who were initially administered warfarin but whose medication changed to NOACs during their treatment period were identified. The follow-up period was from the time of diagnosis of atrial fibrillation to December 31, 2021.

Patients were divided into the warfarin group and the NOAC group based on the administered medication. Common comorbidities of atrial fibrillation and cardiovascular disease, such as hypertension (HTN), diabetes mellitus (DM), dyslipidemia, chronic kidney disease (CKD), peripheral arterial occlusive disease, liver disease, heart failure, and previous myocardial infarctions, were identified prior to the diagnosis of atrial fibrillation using ICD-10 codes of claim data (Supplementary Table 1). Blood test results and blood pressure measurements within 1 year before and after the diagnosis of atrial fibrillation were extracted from the CDW data, and HTN, DM, dyslipidemia, CKD, and liver disease were identified based on these data. HTN was defined as systolic blood pressure ≥140 mmHg or diastolic blood pressure ≥90 mmHg. Blood pressure was checked at the closest time point within 1 year from the diagnosis of atrial fibrillation. An HbA1c of 6.5% or higher was used to diagnose DM. Dyslipidemia was diagnosed as a low-density lipoprotein ≥140 mg/dL. CKD was defined as an estimated glomerular filtration rate (eGFR) <60 mL/min. Liver disease was diagnosed as aspartate aminotransferase, alanine aminotransferase, and alkaline phosphatase >120. The CHA2DS2-VASc and HAS-BLED scores were calculated using the patient data (Supplementary Tables 2, 3). This study was conducted in accordance with the Declaration of Helsinki (as revised in 2013) and approved by the Institutional Review Board of the National Health Insurance Service Ilsan Hospital (NHIMC 2021-07-022). The need for written informed consent was waived as patient identification data were removed from the database used.

### Study outcomes

Four outcome variables were investigated: ischemic stroke, intracranial hemorrhage, gastrointestinal bleeding (GI bleeding), and death. In the CDW+C data, the occurrence of the outcome variables was identified using the corresponding operational definitions and death was determined (Supplementary Table 1). In the CDW-O data, brain magnetic resonance imaging data indicating an ischemic stroke and intracranial hemorrhage were used additionally to identify patients with outcomes was determined.

### Statistical analysis

The analysis period was from the first diagnosis of atrial fibrillation to the occurrence of the outcome variable or the end of the observation period. Continuous data, such as age and blood test results, are presented as mean and standard deviation. Data such as the frequency of comorbidities and the incidence of outcome variables are presented as percentages. We calculated crude incidence as the event numbers by 100 person-years (percentage/years). A chi-square test was conducted to compare and analyze the frequencies of the comorbidities, and a *t*-test was used to compare continuous variables such as age, CHA2DS2-VASc score, and HAS-BLED score. A time-dependent Cox-regression analysis was performed using observed items collected at 95% confidence intervals (CIs) as independent variables to calculate the hazard ratios (HRs) of the occurrence of outcome variables in the warfarin and NOAC groups. Cox proportional regression analysis was performed by including the same independent variables for all outcome variables as a multivariate model. First, it was analyzed by including all comorbidities along with age and sex. Second, when the CHADS2VASC2 score was included, the components of heart failure, HTN, DM, stroke, thromboembolism, and vascular disease were excluded from independent variables. Third, when the HASBLED score was included, the components; HTN, Renal disease, Liver disease, stroke, and prior bleeding were excluded from independent variables. Schoenfeld residuals to check the proportional hazards assumption. For the outcome variable ICH that did not satisfy the proportional hazards assumption, time dependent covariate was added and analyzed. The incidence of each outcome was estimated by use of the Kaplan–Meier estimator. Comparisons between warfarin and NOAC groups were made using a log-rank test. Statistical significance was set at *p* < 0.05 and was two-sided. All statistical analyses were performed using SAS 9.4 (SAS Institute Inc., San Francisco, CA, USA) and R software (version 4.2.0 R Core Team, R Foundation for Statistical Computing, Vienna, Austria).

## Results

**Figure 1:**
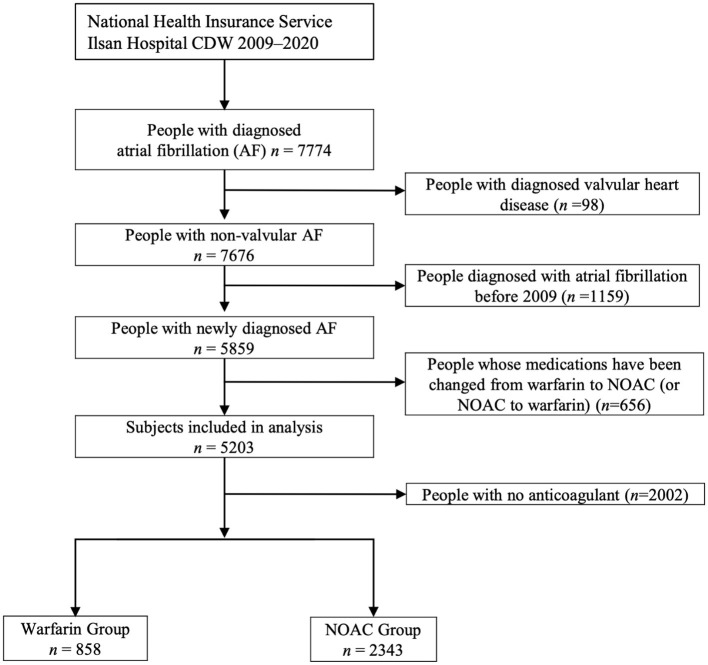
The process of extracting patients with atrial fibrillation from the clinical data warehouse (CDW), combining them with claim data, and selecting study subjects (CDW+C data).

A total of 7,774 patients with atrial fibrillation treated more than once from 2009 to 2020 were identified in the CDW. Ninety-eight (1.26%) patients with valvular disease were excluded from the study. Of the 7,676 patients whose data were extracted from the CDW, 1,159 (15.10%) who were treated for atrial fibrillation at least once before 2009 were excluded. Patients whose medication was changed during the follow-up period were also excluded. Patients who did not receive anticoagulants were excluded. Of the 3,201 (41.7%) patients included in the analysis of CDW+C data, 858 (26.8%) were administered warfarin and 2,343 (73.2%) were administered NOACs (Figure 1). The proportion of patients ≥75 years old was 1,222 (52.2%) in the NOAC group, which was higher than that of the warfarin group (*p* < 0.0001). The rates of previous ischemic stroke, heart failure, and CKD were significantly higher in the warfarin group. The incidence of ischemic stroke, intracranial hemorrhage, GI bleeding, and death were all significantly lower in the NOAC group than in the warfarin group (*p* < 0.0001) (Table 1). The risks of ischemic stroke (HR: 0.479; 95% CI: 0.39–0.589, *p* < 0.0001), intracranial hemorrhage (HR: 0.453; 95% CI: 0.31–0.664, *p* < 0.0001), GI bleeding (HR: 0.579; 95% CI: 0.406–0.824), and all-cause death (HR: 0.502; 95% CI: 0.435–0.58) were lower in the NOAC group than in the warfarin group (Table 2).

**Table 1:** Clinical characteristics and outcomes of patient groups divided according to medications.

	**CDW**−**O dataset**					**CDW**+**C dataset**				
	**Warfarin**	**%**	**NOAC**	**%**	***P-*** **value**	**Warfarin**	**%**	**NOAC**	**%**	***P-*** **value**
*n*	788		1754			858		2343		
Male	429	54.4%	876	49.9%	0.039	453	52.8%	1186	50.6%	0.2748
Age	72.7 ± 10.4		74.3 ± 10.0		<0.0001	70.55 ± 12.43		73.44 ± 10.52		<0.0001
<65	153	19.4%	275	15.7%	<0.0001	247	28.8%	437	18.7%	<0.0001
65~74	253	32.1%	525	29.9%		253	29.5%	684	29.2%	
75~	382	48.5%	954	54.4%		358	41.7%	1222	52.2%	
**Comorbidities**										
Cancer	45	5.7%	126	7.2%	0.172	67	7.8%	243	10.4%	0.0299
Previous ischemic stroke	123	15.6%	158	9.0%	<0.0001	282	32.9%	454	19.4%	<0.0001
Previous intracranial hemorrhage	20	2.5%	38	2.2%	0.567	13	1.5%	32	1.4%	0.7505
Hypertension	552	70.1%	1107	63.1%	0.001	658	76.7%	1750	74.7%	0.2458
Diabetes Mellitus	254	32.2%	415	23.7%	<0.0001	325	37.9%	814	34.7%	0.1006
Dyslipidemia	82	10.4%	211	12.0%	0.254	438	51.0%	1261	53.8%	0.1641
Previous Ischemic heart disease	21	2.7%	50	2.9%	0.798	252	29.4%	638	27.2%	0.2321
Heart failure	157	19.9%	219	12.5%	<0.0001	91	10.6%	162	6.9%	0.0006
Chronic kidney disease	228	28.9%	284	16.2%	<0.0001	111	12.9%	88	3.8%	<0.0001
ESRD	30	3.8%	5	0.3%	<0.0001	65	7.6%	10	0.4%	<0.0001
Peripheral arterial occlusive disease	28	3.6%	49	2.8%	0.317	94	11.0%	320	13.7%	0.0436
Liver failure	61	7.7%	119	6.8%	0.403	78	9.1%	173	7.4%	0.1115
Previous pulmonary thromboembolism	14	1.8%	30	1.7%	0.906	12	1.4%	54	2.3%	0.11
Previous deep vein thrombosis	17	2.2%	21	1.2%	0.077	13	1.5%	26	1.1%	0.3543
Previous systemic thromboembolism	1	0.1%	4	0.2%	0.684	13	1.5%	18	0.8%	0.056
Previous upper GI bleeding	34	4.3%	58	3.3%	0.251	47	5.5%	79	3.4%	0.0066
Previous other GI bleeding	3	0.4%	14	0.8%	0.299	6	0.7%	16	0.7%	0.9603
Previous any bleeding	54	6.9%	143	8.2%	0.263	47	5.5%	172	7.3%	0.0644
CHADS2VASC2 score	3.37 ± 1.61		3.15 ± 1.55		0.001	3.59 ± 1.84		3.59 ± 1.7		<0.0001
HASBLED score	2.60 ± 1.20		2.26 ± 1.12		<0.0001	1.87 ± 0.97		1.83 ± 0.85		0.0025
**Outcome event**										
Ischemic stroke	68	8.6%	60	3.4%	<0.0001	199	23.2%	209	8.9%	<0.0001
Intracranial hemorrhage	25	3.2%	19	1.1%	<0.0001	70	8.2%	61	2.6%	<0.0001
Gastrointestinal bleeding	55	7.0%	83	4.7%	0.023	69	8.0%	78	3.3%	<0.0001
All-Cause death	134	17.0%	130	7.4%	<0.0001	439	51.2%	433	18.5%	<0.0001
BMI (kg/m^*^m) (*n =* 1787)	24.30 ± 3.53	*n =* 443	24.54 ± 3.80	*n =* 850	0.272					
**Laboratory data** ^*^										
Hb (*n =* 1926)	13.04 ± 2.33	*n =* 481	13.20 ± 2.09	*n =* 880	0.187					
Platelet (*n =* 1926)	209.6 ± 74.1	*n =* 481	216.1 ± 70.8	*n =* 880	0.108					
PT (INR) (*n =* 1824)	1.67 ± 1.16	*n =* 596	1.14 ± 0.47	*n =* 742	<0.0001					
NT-pro BNP (*n =* 791)	8529 ± 10564	*n =* 185	4774 ± 6726	*n =* 380	<0.0001					
D-dimer (*n =* 323)	3.78 ± 5.08	*n =* 66	2.85 ± 3.90	*n =* 204	0.117					
free T4 (*n =* 1120)	1.36 ± 0.80	*n =* 271	1.36 ± 0.74	*n =* 506	0.951					
TSH (*n =* 1136)	2.36 ± 3.64	*n =* 278	2.37 ± 5.00	*n =* 511	0.984					
BUN (*n =* 1927)	22.3 ± 15.6	*n =* 480	19.9 ± 11.0	*n =* 888	<0.0001					
Creatinine (*n =* 1928)	1.36 ± 1.42	*n =* 480	0.95 ± 0.47	*n =* 888	<0.0001					
eGFR (CKD-EPI) (*n =* 1928)	65.6 ± 27.7	*n =* 480	72.7 ± 21.2	*n =* 888	<0.0001					
AST (GOT) (*n =* 1789)	44.3 ± 70.8	*n =* 454	43.9 ± 102.0	*n =* 808	0.951					
ALT (GPT) (*n =* 1789)	32.6 ± 55.4	*n =* 454	33.3 ± 58.9	*n =* 808	0.848					
ALP (*n =* 550)	74.4 ± 31.0	*n =* 190	101.4 ± 104.5	*n =* 242	0.001					
Cholesterol (*n =* 966)	151.2 ± 40.5	*n =* 275	159.2 ± 40.7	*n =* 431	0.012					
HDL-Cholesterol (*n =* 881)	39.8 ± 13.9	*n =* 251	46.0 ± 13.2	*n =* 400	<0.0001					
LDL-Cholesterol (*n =* 651)	91.1 ± 33.4	*n =* 195	97.6 ± 30.0	*n =* 294	0.026					
Triglyceride (*n =* 909)	106.0 ± 65.2	*n =* 261	106.7 ± 54.1	*n =* 406	0.894					
HbA1c (*n =* 788)	6.59 ± 1.50	*n =* 230	6.24 ± 1.1	*n =* 370	0.001					
Glucose, AC (*n =* 906)	138.8 ± 64.3	*n =* 297	138.8 ± 64.3	*n =* 358	0.324					

NOAC, non-vitamin K antagonist oral anticoagulant; GI, gastrointestinal; ESRD, end-stage renal disease; PAOD, peripheral arterial occlusive disease; BMI, body mass index; Hb, hemoglobin; PT, prothrombin time; INR, international normalized ratio; NT-pro BNP, N-terminal pro-brain natriuretic peptide; TSH, thyroid stimulating hormone; BUN, blood urea nitrogen; GFR, glomerular filtration rate; CKD-EPI, chronic kidney disease epidemiology collaboration; AST, aspartate amino-transferase; ALT, alanine aminotransferase; ALP, alkaline phosphatase; HDL, high density lipoprotein; LDL, low density lipoprotein. ^*^ n, number of patients with available data.

**Table 2:** Hazards for ischemic stroke, intracranial hemorrhage, GI bleeding and all cause death (CDW+C data).

	**Ischemic stroke**				**Intracranial hemorrhage**			

	**unadjusted HR (95% CI)**	***P-*** **value**	**Adjusted HR (95% CI)**	***P-*** **value**	**unadjusted HR (95% CI)**	***P-*** **value**	**Adjusted HR (95% CI)**	***P-*** **value**
Warfarin	1		1		1		1	
NOAC	0.467 (0.384–0.568)	<0.0001	0.479 (0.39–0.589)	<0.0001	0.452 (0.319–0.639)	<0.0001	0.453 (0.31–0.664)	<0.0001
<65	1		1		1		1	
65~74	0.99 (0.762–1.287)	0.9415	0.997 (0.755–1.397)	0.9853	1.554 (0.983–2.457)	0.0593	1.833 (1.105–3.039)	0.0189
75~	1.257 (0.983–1.608)	0.0679	1.248 (0.943–1.65)	0.1207	1.365 (0.855–2.179)	0.1926	1.574 (0.919–2.697)	0.0985
CHADS2VASC2 score	1.089 (1.03–1.151)	0.0028	1.096 (0.997–1.205)	0.0582	1.209 (1.095–1.336)	0.0002	*	
HASBLED score	1.022 (0.92–1.136)	0.6817	*		1.363 (1.133–1.64)	0.001	1.139 (0.901–1.438)	0.2765
	**GI bleeding**				**All cause death**			
Warfarin	1		1		1		1	
NOAC	0.573 (0.413–0.795)	0.0009	0.579 (0.406–0.824)	0.0024	0.501 (0.438–0.573)	<0.0001	0.502 (0.435–0.58)	<0.0001
<65	1		1		1		1	
65~74	1.49 (0.883–2.514)	0.1348	1.46 (0.844–2.523)	0.1759	2.328 (1.762–3.076)	<0.0001	2.549 (1.912–3.399)	<0.0001
75~	3.083 (1.917–4.96)	<0.0001	3.045 (1.799–5.153)	<0.0001	7.096 (5.504–9.15)	<0.0001	7.746 (5.902–10.166)	<0.0001
CKD	2.691 (1.597–4.533)	0.0002	1.287 (0.596–2.782)	0.5209	3.191 (2.61–3.902)	<0.0001	1.546 (1.168–2.046)	0.0023
CHADS2VASC2 score	1.3 (1.181–1.43)	<0.0001	0.994 (0.841–1.176)	0.9469	1.418 (1.363–1.475)	<0.0001	1.09 (1.019–1.165)	0.0123
HASBLED score	1.691 (1.421–2.012)	<0.0001	1.572 (1.256–1.968)	<0.0001	1.586 (1.476–1.704)	<0.0001	1.145 (1.034–1.268)	0.0091

NOAC, non-vitamin K antagonist oral anticoagulant; HR, hazard ratio; CI, confidence intervals; GI, gastrointestinal; CKD, chronic kidney disease. Adjusted for age, sex and comorbidities. ^*^not included as an adjustment variable.

**Figure 2:**
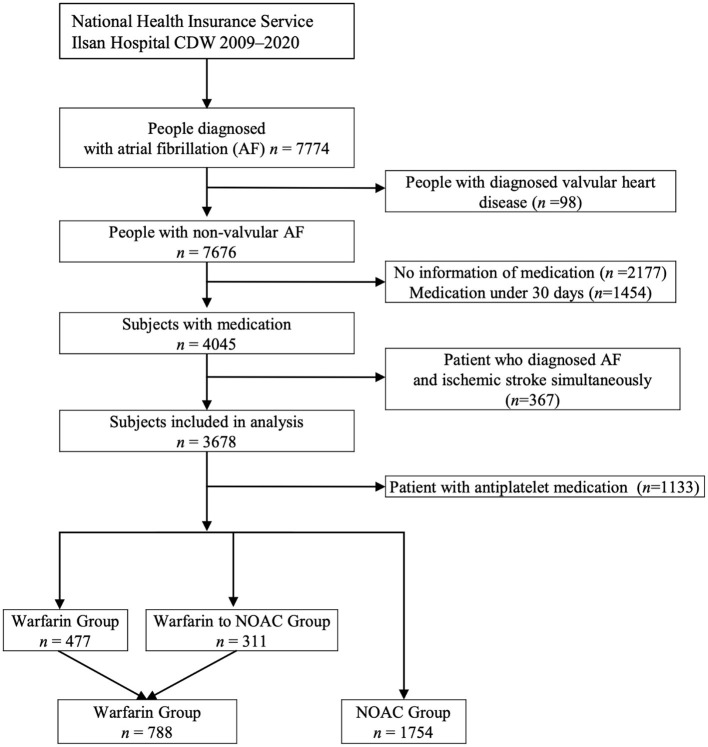
The process of extracting patients with atrial fibrillation from the clinical data warehouse (CDW) and selecting subjects for the analysis (CDW-O data).

**Figure 3:**
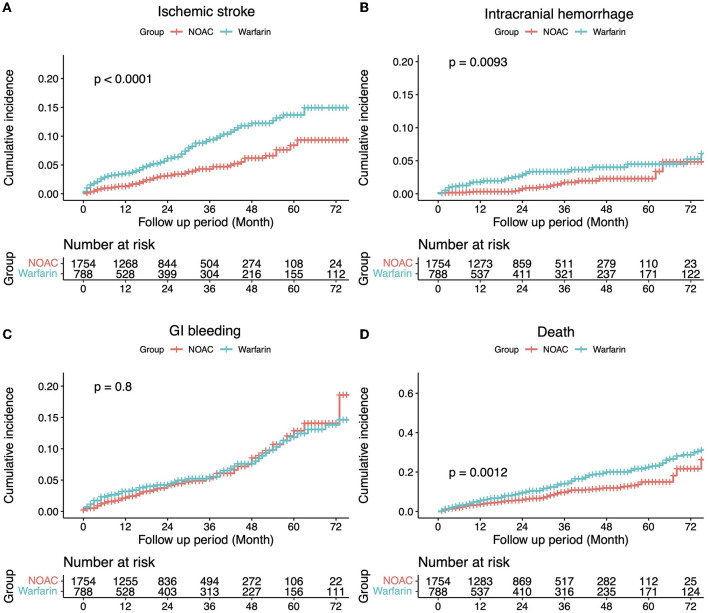
The incidence of each outcome was estimated using the Kaplan–Meier estimator. Comparisons between groups were made using a log-rank test **(A)** Ischemic stroke, **(B)** Intracranial hemorrhage, **(C)** Gastrointestinal bleeding, and **(D)** Death.

Of the 7,676 patients, 2,542 (33.12%) had sufficient clinical data and were included in the analysis of CDW-O data. The warfarin group included 788 (31.00%) patients and the NOAC group included 1,754 (69.00%) patients (Figure 2). As in the CDW+C data, the mean age of the warfarin group was significantly lower than that of the NOAC group (*p* < 0.0001). A total of 153 (19.4%) of patients in the warfarin group were <65 years of age, which was significantly lower than that of the NOAC group (15.7%) (*p* < 0.0001). As in the CDW+C data, the rates of previous ischemic stroke, heart failure, and CKD, and additionally HTN and DM were significantly higher in the warfarin group than in the NOAC group. The mean CHA2DS2-VASc (*p* = 0.001) and HAS-BLED (*p* < 0.0001) scores were significantly higher in the warfarin group than in the NOAC group. In the warfarin group, the mean international normalized ratio (INR) was 1.67, the mean eGFR was 65.6 mL/min, and the mean HbA1c was 6.59%. The INR of the warfarin group were statistically greater than those in the NOAC group due to drug effect (*p* < 0.0001). Regarding the higher comorbidities of DM and CKD, HbA1c in the warfarin group was higher and eGFR in the warfarin group was lower than those in the NOAC group significantly (*p* = 0.001, *p* < 0.0001). The median follow-up period was 2.3 years (interquartile range 0.8-4.9 years) in warfarin group and 2.4 years (interquartile range 1.2-4.2 years) in the NOAC group. The outcome events were identified at a lower rate than the CDW+C data, except for GI bleeding. All outcome events occurred significantly lower in the NOAC group than in the warfarin group, as in the CDW+C data (Table 1). The incidence of ischemic stroke, intracranial hemorrhage and GI bleeding was 2.75, 1.01, and 2.22/100 person-years in the warfarin group and 1.14, 0.36, and 1.57/100 person-years in the NOAC group, respectively. The mortality was 5.42/100 person-years in the warfarin group and 2.47/100 person-years in the NOAC group. The NOAC group had lower risks of ischemic stroke (HR: 0.399; 95% CI: 0.282–0.565), intracranial hemorrhage (HR: 0.430; 95% CI: 0.236–0.785), and death (HR: 0.671; 95% CI: 0.515–0.873) than the warfarin group (Table 3). The Kaplan-Meier survival curve demonstrated a significant higher cumulative incidence of stroke and death in the warfarin group (Figure 3).

**Table 3:** Hazards for ischemic stroke intracranial hemorrhage, GI bleeding and cardiovascular death (CDW-O data).

	**Ischemic stroke**				**Intracranial hemorrhage**			

	**Unadjusted HR (95% CI)**	***P-*** **value**	**Adjusted HR (95% CI)**	***P-*** **value**	**unadjusted HR (95% CI)**	***P-*** **value**	**Adjusted HR (95% CI)**	***P-*** **value**
Warfarin	1		1		1		1	
NOAC	0.401 (0.284–0.568)	<0.0001	0.399 (0.282–0.565)	<0.0001	0.380 (0.212–0.684)	0.001	0.430 (0.236–0.785)	0.006
<65	1		1		1		1	
65~74	1.654 (1.293–2.117)	<0.0001	1.505 (0.860–2.635)	0.152	2.228 (1.543–3.216)	<0.0001	0.666 (0.299–1.481)	0.319
75~	2.017 (1.6–2.544)	<0.0001	2.037 (1.191–3.483)	0.009	1.925 (1.329–2.786)	0.0005	0.608 (0.270–1.368)	0.229
CKD	1.371 (0.901–2.086)	0.14	0.985 (0.625–1.551)	0.947	2.947 (1.614–5.379)	<0.0001	2.258 (1.214–4.199)	0.01
CHADS2VASC2 score	1.145 (1.028–1.276)	0.014	0.997 (0.849–1.170)	0.97	1.188 (0.992–1.424)	0.062	1.236 (0.975–1.567)	0.08
HASBLED score	1.288 (1.111–1.493)	0.001	1.223 (1.055–1.419)	0.008	1.335 (1.043–1.709)	0.022	1.330 (1.004–1.763)	0.047
	**GI bleeding**				**All cause death**			
Warfarin	1		1		1		1	
NOAC	0.788 (0.564–1.102)	0.164	0.787 (0.555–1.117)	0.18	0.655 (0.506–0.848)	0.001	0.671 (0.515–0.873)	0.003
<65	1		1		1		1	
65~74	1.654 (1.293–2.117)	<0.0001	1.370 (0.720–2.607)	0.337	1.717 (0.966–3.052)	0.065	1.557 (0.873–2.779)	0.134
75~	2.017 (1.6–2.544)	<0.0001	3.357 (1.858–6.065)	<0.0001	5.281 (3.119–8.941)	<0.0001	4.479 (2.627–7.638)	<0.0001
CKD	2.142 (1.498–3.061)	<0.0001	1.612 (1.119–2.322)	0.01	3.055 (2.388–3.907)	<0.0001	2.070 (1.600–2.677)	<0.0001
CHADS2VASC2 score	1.336 (1.208–1.477)	<0.0001	1.097 (0.949–1.268)	0.21	1.337 (1.238–1.443)	<0.0001	1.106 (0.995–1.229)	0.062
HASBLED score	1.423 (1.237–1.635)	<0.0001	1.260 (1.079–1.470)	0.003	1.432 (1.294–1.585)	<0.0001	1.249 (1.101–1.416)	0.001

NOAC, non-vitamin K antagonist oral anticoagulant ; HR, hazard ratio; CI, confidence intervals; GI, gastrointestinal; CKD, chronic kidney disease. Adjusted for age, sex and comorbidities.

## Discussion

In this study, two datasets (CDW+C and CDW-O) were constructed by using CDW and claim data to identify clinical outcomes. The results of each dataset in this study were similar. Patients administered NOACs had a lower risk of ischemic stroke, intracranial hemorrhage, and death than those administered warfarin. The risk of GI bleeding was not statistically lower in the NOAC group than in the warfarin group in the CDW-O data but was statistically lower in the CDW+C data. CKD was shown to increase the risk of intracranial hemorrhage, GI bleeding, and death in the CDW-O data. CKD is a major risk factor for cardiovascular disease and death and increases the bleeding risk (18). However, patients with CKD may have been identified more accurately in the CDW-O data than in the CDW+C data, which may have contributed to these results. The HAS-BLED score was associated with the risk of developing GI bleeding in both datasets.

The findings of this study are consistent with those of previous studies. The first study comparing and analyzing ischemic stroke, intracranial hemorrhage, and all-cause death between warfarin and NOACs based on claim data in Korea found that patients administered NOACs had a similar risk of developing ischemic stroke as patients administered warfarin, and the cause of intracranial hemorrhage and all-cause death was lower in the NOAC group (15). A follow-up study reported that patients administered NOACs have a lower risk of ischemic stroke and GI bleeding than those who were administered warfarin (19).

In this study, more clinical outcomes were identified in the CDW+C data than in the CDW-O data. Ischemic stroke occurred in 8.6% of patients in the warfarin group and 3.4% of patients in the NOAC group in the CDW-O data and in 23.2% of patients in the warfarin group and 8.9% of patients in the NOAC group in the CDW+C data. Although these differences are significant, the ratio of ischemic stroke in the two groups is similar in each dataset. Intracranial hemorrhage occurred in 3.2% of patients in the warfarin group and 1.1% of patients in the NOAC group in the CDW-O data and in 8.2% of patients in the warfarin group and 2.6% of patients in the NOAC group in the CDW+C data, which was a similar ratio between the two groups in each dataset. GI bleeding occurred in 7.1% of patients in the warfarin group and 5.1% of patients in the NOAC group in the CDW-O analysis and in 8.0% of patients in the warfarin group and 3.3% of patients in the NOAC group in the CDW+C data. These values were similar between the two datasets.

In previous claim data studies, the annual incidence of ischemic stroke was ~1.5% in both the warfarin and NOAC groups during a 2-year follow-up period and 2.96% in the warfarin group and 2.07–2.36% in the NOAC group in a subsequent follow-up study (15, 19). The median follow-up period of in these past studies were 0.8 years, but we followed a longer period, median 2.3–2.4 years, in this study. The incidence of ischemic stroke in our study was lower than that of previous study, and the difference was confirmed to be greater in the warfarin group. The CDW-O data does not reflect the medical records of patients treated at other hospitals. However, since the occurrence of stroke was determined based on magnetic resonance imaging results, the definition of stroke in this analysis may be more accurate than the occurrence of stroke based on the operational definition. The incidence of ischemic stroke in previous studies may have been overestimated if based on the operational definition of the claim data, whereas the incidence in this study may have been underestimated as data from other hospitals was not available in the CDW-O data.

In the CDW+C data, the follow-up period of the NOAC group was a maximum of 4 years and 6 months, and ischemic stroke occurred in 8.9% of patients, which was consistent with the incidence of ischemic stroke reported in a previous study (19). This may be because the studies used the same operational definition for ischemic stroke. The incidence of ischemic stroke is higher in the warfarin group in this study as the follow-up period of the warfarin group is longer than that of the NOAC group. The warfarin group had a maximum follow-up period of 12 years, and ischemic stroke occurred in 23.2% of patients. As the annual incidence rate of ischemic stroke in the warfarin group has been reported as 2.61%, the incidence rate in this study should not be considered to be high. In addition, after chronic warfarin use, the INR deviates from the treatment target range, which may increase the incidence of ischemic stroke. Among our study subjects, the number of patients whose INR was adjusted within the target range was 141 (23.7%), and 182 (30.5%) including the subtherapeutic range (INR > 1.7). Although the risk of ischemic stroke was higher in patients whose INR was not within the target range, statistical significance could not be confirmed due to the small number of patients. As the incidences of GI bleeding were based on the same operational definitions in the CDW-O and CDW+C data in this study, the differences in the results of the analyses may be because the CDW+C data included the treatment history of the patients treated at other hospitals and a longer follow-up period.

In this study, the warfarin group had more comorbidities than the NOAC group. In the CDW-O data, HTN and DM were identified at a higher rate in the warfarin group than in the NOAC group, but in the CDW+C data, there was no difference in the rate of HTN and DM between the two groups. Since many patients were treated in primary care institutions for HTN and DM, discrepancies may have occurred in the CDW+O data, which is based on the EMR of referral hospital. In the CDW+C data, which combines the treatment history of all medical institutions, there was no difference in the rate of HTN and DM as comorbidities, and it is thought to be close to the actual clinical data. Old ischemic stroke was confirmed about 1.7 times more in the warfarin group than the NOAC group in both CDW-O and CDW+C data. However, old ischemic stroke was not a significant predictor of ischemic stroke after diagnosis of atrial fibrillation. Among the comorbidities, dyslipidemia and CKD showed the largest difference between the CDW+C and CDW-O datasets. Since dyslipidemia drugs are frequently prescribed in primary care clinics other than in our hospital, from where CDW data were extracted, dyslipidemia can be accurately identified at a higher rate in CDW+C data. CKD was more frequent in the CDW-O data than in the CDW+C data. The rate of CKD in the CDW-O data may be more accurate as it was based on the results of blood tests. Patients with stage 3 CKD may have been difficult to identify using claim data. A claim data study conducted in other Asian countries reported that CKD occurred in 20–29% of patients, which is similar to the results of the CDW-O data in this study (20).

This study has several limitations. First, although the patients' clinical information was obtained from the CDW, several patients had missing information. The patients' test results were used to account for the missing comorbidity data. Second, the NOAC group could not be analyzed further based on the four different medications due to a small patient population. Larger multi-center studies should be conducted. Last, a selection bias cannot be ruled out in this study as the patients were from a single institution in one country, which may have resulted in a higher incidence of clinical outcomes. Future studies with similar follow-up periods should be conducted using claim data.

## Conclusion

In conclusion, this study combined data from the CDW with claim data, resulting in more accurate clinical findings. The findings indicated that NOACs are more effective than warfarin for the prevention of ischemic stroke and reduction of hemorrhagic side effects in patients with atrial fibrillation at long-term follow-up. Patients with atrial fibrillation should be treated with NOACs to reduce the incidence of ischemic stroke.

## Data availability statement

The raw data supporting the conclusions of this article will be made available by the authors, without undue reservation.

## Ethics statement

The studies involving human participants were reviewed and approved by the Institutional Review Board of the National Health Insurance Service Ilsan Hospital. Written informed consent for participation was not required for this study in accordance with the national legislation and the institutional requirements.

## Author contributions

JH: organized the database and performed the statistical analysis. HL: clinical data collection, interpretation, and collection of cardiac parameter data. K-DS: conceptualization, methodology, investigation, resources, data curation, supervision, and manuscript writing. All authors have read and approved the final manuscript.
