# Supplementary material for: Long-term stroke and major bleeding risk in patients with non-valvular atrial fibrillation: A comparative analysis between non-vitamin K antagonist oral anticoagulants and warfarin using a clinical data warehouse
Source: Front Neurol. 2023 Jan 26;14:1058781. doi: 10.3389/fneur.2023.1058781 (PMC9923348; doi:10.3389/fneur.2023.1058781)
Supplement: Supplementary file 1 [file Table_1.DOCX]

Supplementary materials

| Table 1. Definition of comorbidities and outcomes | | | | |
| --- | --- | --- | --- | --- |
| Diagnosis | | ICD-10-CM code and definition | Diagnoses |  |
| Hypertension | | I10–I13, I15 | Patients had ≥1 diagnosis during hospitalization or ≥2 diagnoses at outpatient clinic for preventing overestimation of diagnosis. |  |
| Diabetes mellitus | | E11–E14 |  |  |
| Dyslipidemia | | E78 |  |  |
| CKD (Renal disease) | | N18, N19 (I13.1, N03, N05, N10-19, Z49, Z94.0, Z99.2) |  |  |
| Liver disease (including cirrhosis) | | K70, K72-76, K71.3-K71.7 |  |  |
| ESRD |  | N185, Z49; dialysis ≥2 |  |  |
| Heart failure | | I50 | Patients had one or more diagnoses during hospitalization or at outpatient clinic. |  |
| Venous thromboembolism | | I26 (pulmonary thromboembolism) |  |  |
|  | | I80-I82 (deep vein thrombosis) |  |  |
| Prior systemic thromboembolism | | I74 |  |  |
| Vascular disease | |  |  |  |
|  | Prior myocardial infarction | I21, I22 |  |  |
|  | Peripheral artery disease | I70, I73 |  |  |
| Prior ICH |  | I60, I61, 62, S064, S065, S066 | Patients had ≥1 diagnoses during hospitalization. And CT, RBC transfusion |  |
| Prior ischemic stroke | | I63 | Patients had ≥1 diagnoses during hospitalization. And CT, MRI |  |
| Prior transient ischemic attack | | G458, G459 | Patients had ≥1 diagnoses during hospitalization. And CT, MRI |  |
| Prior GI bleeding |  |  |  |  |
|  | upper GI bleeding | K250, K252, K254, K256, K260, K262, K264, K266, K270,  K272, K274, K276, K280, K282, K284, K286, K290, K920-922 | Patients had ≥1 diagnoses during hospitalization and packed RBC transfusion ≥1 (packed RBC code: X2021, 2022, 2031, 2032, 2091, 2092, 2111, 2112, 2131, 2132) |  |
|  | other GI bleeding | I850, K226, K552, K625, K633, K649 |  |  |
| Prior any bleeding | bleeding from other sites | D500, D62, D683, D698, D699, H052, H113, H210, H313, H357, H431, H448, H470, M250, N421, N831, N857, N920, N923, N930, N938, N939, R040, R041, R042, R048, R049, R233, R310, R311, R318, R58, T792, T810 |  |  |
| Cancer |  | C00-97 and cancer code (V193) |  |  |
| Outcome |  |  |  |  |
| Major bleeding |  |  |  |  |
|  | ICH | I60, I61, I62 | Patients had ≥1 diagnoses during hospitalization and packed RBC transfusion ≥1 (code: X2021, 2022, 2031, 2032, 2091, 2092, 2111, 2112, 2131, 2132) |  |
|  | GI bleeding | K260, K262, K264, K266, K270, K272, K274, K276, K280, K282, K284,  K286, K290, K625, K920, K921, K922 |  |  |
| Ischemic stroke |  | I63 | Patients had ≥1 diagnoses during hospitalization and MRI code |  |
| Death |  |  | all-cause mortality |  |

CKD, chronic kidney disease; ESRD, end-stage renal disease; ICH, intracerebral hemorrhage; RBC, red blood cell; GI, gastrointestinal.

| Table 2. Scoring and International Classification of Disease 10th Revision (ICD-10) codes for the factors included in the CHA2DS2-VASc score | | | |
| --- | --- | --- | --- |
| Condition | ICD-10 codes | Point |  |
| Congestive heart failure | I50 | 1 |  |
| Hypertension | I10–I13, I15 | 1 |  |
| Age | ≥75 years | 2 |  |
| Diabetes | E11–E14 | 1 |  |
| Stroke or systemic embolism | I63, G458, G459, I74, I26, I80-82 | 2 |  |
| Vascular disease | I21, I22, I70, I73 | 1 |  |
| Age | 65–74 years | 1 |  |
| Sex | Female | 1 |  |
|  |  |  |  |
| Table 3. Scoring and International Classification of Disease 10th Revision (ICD-10) codes for the factors included in the HAS-BLED score | | | |
| Condition | ICD-10 codes | Point |  |
| Hypertension | I10–I13, I15 | 1 |  |
| Abnormal renal disease | N18, N19 (I13.1, N03, N05, N10-19, Z49, Z94.0, Z99.2), N185, Z49; dialysis ≥2 | 1 |  |
| Abnormal liver function | K70, K72-76, K71.3-K71.7 | 1 |  |
| Stroke | I63, G458, G459 | 1 |  |
| Bleeding history or predisposition* | D500, D62, D683, D698, D699, H052, H113, H210, H313, H357, H431, H448, H470, M250, N421, N831, N857, N920, N923, N930, N938, N939, R040, R041, R042, R048, R049, R233, R310, R311, R318, R58, T792, T810, K250, K252, K254, K256, K260, K262, K264, K266, K270, K272, K274, K276, K280, K282, K284, K286, K290, K920-922, I850, K226, K552, K625, K633, K649, I60, I61, 62, S064, S065, S066 | 1 |  |
| Elderly | ≥65 years | 1 |  |
| Drug therapy | Antiplatelets and NSAIDs | 1 |  |
| Alcoholism | E244, F10, G312, G621, G721, I426, K292, K70, K860, O354, P043, Q860, T510, X45, X65, Y15, Y90-Y91, Z502, Z714 and Z721 | 1 |  |
| * A blood transfusion was also required to define a history of bleeding from other sites. | | |  |
